# A Novel Conductive Antibacterial Nanocomposite Hydrogel Dressing for Healing of Severely Infected Wounds

**DOI:** 10.3389/fchem.2021.787886

**Published:** 2021-11-24

**Authors:** Lizhi Xiao, Fang Hui, Tenghui Tian, Ruyue Yan, Jingwei Xin, Xinyu Zhao, Yingnan Jiang, Zhe Zhang, Yulan Kuang, Na Li, Yu Zhao, Quan Lin

**Affiliations:** ^1^ Jilin Ginseng Academy, Hospital of Affiliated Changchun University of Chinese Medicine, Changchun University of Chinese Medicine, Changchun, China; ^2^ State Key Laboratory of Supramolecular Structure and Materials, College of Chemistry, Chinese-Japan Union Hospital of Jilin University, Jilin University, Changchun, China; ^3^ Key Laboratory of Songliao Aquatic Environment of Ministry of Education, Jilin Jianzhu University, Changchun, China

**Keywords:** conductivity, Ag nanoparticles, bacteriostatic medical hydrogel, infected wound healing, new animal model

## Abstract

Wound infections are serious medical complications that can endanger human health. Latest researches show that conductive composite materials may make endogenous/exogenous electrical stimulation more effective, guide/comb cell migration to the wound, and subsequently promote wound healing. To accelerate infected wound healing, a novel medical silver nanoparticle-doped conductive polymer-based hydrogel system (Ag NPs/CPH) dressing with good conductivity, biocompatibility, and mechanical and antibacterial properties was fabricated. For the hydrogel dressing, Ag NPs/CPH, polyvinyl alcohol (PVA), and gelatin were used as the host matrix materials, and phytic acid (PA) was used as the cross-linking agent to introduce conductive polyaniline into the matrix, with antibacterial Ag NPs loaded via impregnation. After a series of analyses, the material containing 5 wt% of PVA by concentration, 1.5 wt% gelatin, 600 μL of AN reactive volume, and 600 μL of PA reactive volume was chosen for Ag NPs/CPH preparation. XPS and FTIR analysis had been further used to characterize the composition of the prepared Ag NPs/CPH. The test on the swelling property showed that the hydrogels had abundant pores with good water absorption (≈140% within 12 h). They can be loaded and continuously release Ag NPs. Thus, the prepared Ag NPs/CPH showed excellent antibacterial property with increasing duration of immersion of Ag NPs. Additionally, to evaluate *in vivo* safety, CCK-8 experiments of HaCat, LO2 and 293T cells were treated with different concentrations of the Ag NPs/CPH hydrogel soaking solution. The experimental results showed the Ag NPs/CPH had no significant inhibitory effect on any of the cells. Finally, an innovative infection and inflammation model was designed to evaluate the prepared Ag NPs/CPH hydrogel dressing for the treatment of severely infected wounds. The results showed that even when infected with bacteria for long periods of time (more than 20 h), the proposed conductive antibacterial hydrogel could treat severely infected wounds.

## 1 Introduction

Wound infections refer to the serious inflammatory reaction that occurs after a relatively long period of time (more than 24 h) when microorganisms invade damaged skin (Brook and Frazier, 1990; Brook and Frazier, 1998). The clinical manifestations include local swelling, pain, pus exudation, and a peculiar smell. Serious wound infections may cause a systemic infection, which can be fatal. Thus, it is imperative to cure infected wounds and accelerate healing. Traditional wound dressings, such as gauze and bandages, have good air permeability but do not have bacteriostatic effects on the infected wounds (Catanzano et al., 2021; Chi et al., 2020; Niimi et al., 2020; dos Santos et al., 2018; Varaprasad et al., 2020; Zhang and Zhao, 2020). During treatment, secondary injuries can easily occur due to wound adhesion, increasing the wound healing cycle time, and resulting in scarring. Recently, various new antibacterial dressings such as films, foams, and alginate have been developed to address the clinical needs of wound healing. However, these materials have some disadvantages such as lack of osmotic absorption, poor wound surface adhesion, and low air permeability. Thus, a novel, light, and portable medical dressing is needed that can effectively resist bacteria and promote the healing of infected wounds.

Hydrogels are three-dimensional (3D) polymer networks that can swell and absorb large quantities of water. Unlike other dressings, hydrogel wound dressings can provide a benign moist environment suitable for wound healing, accelerate granulation and blood vessel generation, and promote the self-dissolution of necrotic tissue (Song Y. et al., 2021). In addition, exudate, which can promote infections, can be easily absorbed or exchanged by the hydrogel material (Koehler et al., 2018). Because of these characteristics, compound medical hydrogel dressings are good prospects for clinical applications (Cohen et al., 2001; Jridi et al., 2015; Francesko et al., 2017; Shanmugapriya et al., 2020).

Polyvinyl alcohol (PVA) has been widely used in medical biomaterials due to its lubricating properties, elasticity, and shock absorption abilities (Stammen et al., 2001; Holloway et al., 2013; Ding et al., 2018; Lotfipour et al., 2019; Zhou et al., 2019). It also has good mechanical properties when compounded with other suitable materials. Zhou et al. (2019) incorporated hydroxypropyl cellulose (HPC) into physically crosslinked PVA, forming HPC/PVA hydrogels with high mechanical strengths and strong ionic conductivities. Ding et al. (2018) also developed a composite PVA-borax gel matrix by using nanostructured materials, and the material exhibited electrical conductivity, self-healing capabilities, biocompatibility, viscoelasticity, and had good mechanical properties. Recently, a series of diverse PVA hydrogels, formed under freeze-thaw cycles, have attracted attention (Kenawy et al., 2010; Holloway et al., 2013; Lotfipour et al., 2019). For example, Holloway et al. (2013) studied the differences in mechanical properties of PVA hydrogels during freeze-thaw cycles In addition, Lotfipour et al. (2019) used PVA and chitosan as substrates, along with oxytetracycline to prepare and evaluate a medical hydrogel formed after freeze-thaw cycling.

Gelatin contains numerous active functional groups such as amino, carboxyl, and hydroxyl groups. It has suitable characteristics such as high-water absorption, low antigenicity, good biocompatibility, and biodegradability (Doi et al., 2007; Ooi et al., 2015; Yang et al., 2020; Chen et al., 2021). In addition, as the hydrolysate of natural polymer collagen, gelatin-based medical hydrogels have shown potential in promoting angiogenesis (Doi et al., 2007). However, the mechanical properties and thermal stability of hydrogels synthesized with these natural polymers are poor, which significantly limits their applications. Therefore, by using a combination of gelatin and other materials to prepare interpenetrating networks, dual network and nanocomposite hydrogels with better mechanical properties have become a trending research topic.

With the rapid development of artificial intelligence and micro-robotics, medical hydrogels have broad application prospects for biomedical sensing, due to their stretchability, wearability, and drug delivery applications (Pan et al., 2012; Zhai et al., 2013; Hasanzadeh et al., 2014; Bagheri et al., 2020; Chu et al., 2020; Xu et al., 2020). Previous studies have shown that medical hydrogel dressings can mold to the skin, as they are conducive to electrical signals (Hasanzadeh et al., 2014; Xu et al., 2020).

Of the many conductive polymer materials, polyaniline (PANI) exhibits good conductivity, has a simple modification process, and can be easily combined with other hydrogel materials (Guimard et al., 2007; Pan et al., 2012; Zhai et al., 2013; Bagheri et al., 2020; Chu et al., 2020; Song et al., 2020). Thus, this material has been extensively studied for bioelectrical sensing and electrochemical detection (Pan et al., 2012; Zhai et al., 2013). For example, Song et al. (2020) coated PANI on polycarboxylic and polybranched cellulose nanocrystal surfaces to prepare multi CNC-PANI. Then, the multi CNC-PANI was added to the PVA/borax doped hydrogel structure and a mechanical, stretchable, sensitive, and self-healing hydrogel with skin-like properties was obtained. In addition, reports have shown that doped PANI can be used to prepare antibacterial materials to prevent the growth of bacteria and other microorganisms (Guimard et al., 2007). The latest research show that conductive composite materials may make endogenous/exogenous electrical stimulation more effective and guide/comb cell migration to the wound. Thus, it can promote the cell activity of wound healing, as well as help to monitor the progress of healing (Korupalli et al., 2020; Wu et al., 2021). However, few reports have investigated PANI composite hydrogel materials with both conductive and antibacterial properties.

Phytic acid (PA) is an organic phosphorus compound that is extracted from plants and contains six non-coplanar phosphate bonds. As a cross-linking agent/dopant, PA is widely used in food and drug manufacturing, metal smelting, and the petrochemical industry. PA is also one of the most common crosslinking agents for composite PANI materials, and it can crosslink PANI and other conductive materials into a conductive 3D network structure (Pan et al., 2012; Zhai et al., 2013). For example, Pan et al. (2012) used PANI cross-linked with phytic acid to prepare hydrogel films with good electrochemical properties.

In addition, drug-resistant bacteria are increasing due to the overuse of antibiotics. Thus, medical hydrogel dressings that incorporate antibacterial agents, other than antibiotics, are of significant interest (Hernando-Amado et al., 2019). Among these agents, silver nanoparticles (Ag NPs) have been extensively used as antibacterial materials. Ag NPs can change the permeability of the bacterial cell membrane, while the silver ions damage bacterial DNA, thus reducing dehydrogenase activity. Because of their disinfecting properties, Ag NPs have been extensively used in antibacterial treatments such as doping hydrogel applicators, without increasing the prevalence of drug-resistant bacteria (Xiu et al., 2012; Mao et al., 2017; Song et al., 2021). Compared with ordinary carriers, the nanocomposite hydrogel material can slowly release silver nanoparticles to the infected wound surface and achieve a long-term antibacterial effect (Palem et al., 2019; Jiang et al., 2020).

In a clinical setting, the wound may become seriously infected and purulent without timely treatment, and can become life-threatening. Typically, when studying antimicrobial gel dressings, the environment is clean (generally SPF grade), and the researchers only conduct simple bacteria-smear tests and observe the wound healing process. For example, the antibacterial dressing is immediately applied after coating the wound surface with bacteria (Wang et al., 2017; Zhao et al., 2017; Chen et al., 2018; Lei and Wu, 2018; Chadwick and Ousey, 2019; Liang et al., 2019; Arnold, 2020; Liang et al., 2021; Lu et al., 2021). As a result, wound infection is typically mild in these types of experiments. However, to the best of our knowledge, other studies on bacteriostatic hydrogel applicators have not considered simulating and treating severe wound infections.

**SCHEME 1:**
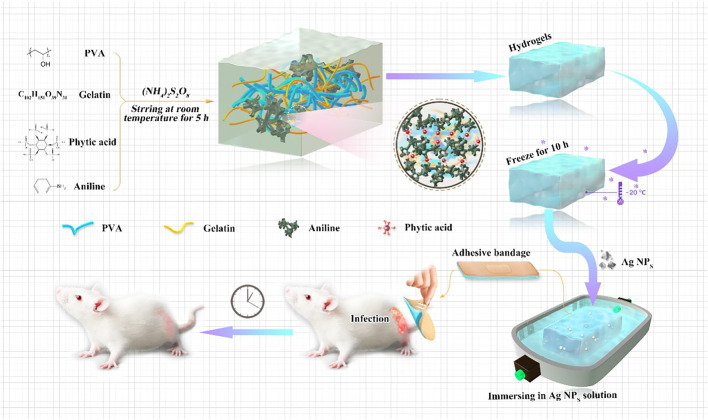
Synthesis process of the Ag NPs/CPH medical hydrogel, and its applications in a new animal model for infected wounds.

In this work, we synthesized a novel hydrogel with good mechanical properties and bacteriostatic effects, and we investigated its therapeutic properties on severely infected wounds. PVA (5 wt%) and gelatin (1.5 wt%) were used as the main matrix materials of the composite hydrogel. In addition, conductive PANI with PA as the crosslinking agent was used to provide conductivity in the conductive polymer-based hydrogel system (CPH). To improve the antibacterial properties of the hydrogel CPH, Ag NPs protected by polyvinylpyrrolidone with good antibacterial properties were loaded by soaking. Thus, a new conductive skin, fitted with medical dressing (Ag NPs/CPH) and good antibacterial properties, was obtained. Of note, we also created an animal model for severe wound infections. We confirmed that the prepared antibacterial Ag NPs/CPH hydrogel dressing prepared in this study had good bacteriostatic effects on infected wounds that were infected for 48 h with *staphylococcus aureus* (*S. aureus*). In addition, we conducted a series of analyses on the new Ag NPs/CPH hydrogel dressing, confirming its good mechanical properties, conductivity, biocompatibility, and bacteriostatic effects on infected wounds (Scheme 1). Thus, a new medical gel applicator for severe wound infections was fabricated.

## 2 Experimental Section

### 2.1 Materials

Aniline (AN, ≈ 99.5%) and phytic acid (PA, ≈ 50 wt%, Mw ≈ 660.04) were purchased from Aladdin; polyvinylpyrrolidone (PVP, Mw ≈ 40,000), PVA (≈99% hydrolyzed, Mw ≈ 130,000), and gelatin (≈300 g, bloom) were purchased from Sigma-Aldrich; silver nitrate (AgNO_3_, ≈99.8%), and sodium borohydride (NaBH_4_, ≈96%) were purchased from Sinopharm Chemical Reagent Co., Ltd (Shanghai, P. R. China); ammonium persulfate (APS, ≈ 98.5%), hydrogen peroxide (H_2_O_2_, 30 wt%), and sodium citrate (≈99.0%) were obtained from Beijing Chemical Works (Beijing, P. R. China).

### 2.2 Devices

X-ray photoelectron spectroscopy (XPS) was performed using a Thermo ESCALAB 250. The radiation source is monochromatic Al Ka (hv = 1,486.6 eV). The passing energy is 20 eV C1s (284.6 eV) is used for calibration. The vacuum exerts more than 2 × 10^−6^ Pa of pressure. The FTIR spectra of the raw materials and hydrogels prepared are characterized on Fourier transform infrared spectroscopy BRUKER VECTOR33. Scanning electron microscope (SEM) images were obtained using a JEOL FESEM6700F electron microscope with a primary electron energy of 3 kV. Transmission electron microscopy (TEM) images of the Ag NPs were obtained using a Tecnai GI F20 U-TWIN. The tensile data were obtained using electronic universal material testing machine Instron 5,982. The OD values of bacteria and cells were determined with an Infinite 200 Pro microplate reader. The image of hematoxylin-eosin staining (H&E) was obtained from the electron microscope CIC XSP-C204. All experimental water was purified using an ultrapure water machine WP-UP-YJ-40. The dielectric constant was determined using an Agilent Precision Impedance Analyzer 4294A. The dynamic storage modulus (E′) and loss modulus (E″) were executed (0–50 Hz) using a dynamic thermomechanical analyzer DMA+450. The rheometer was purchased from TA model DHR-2. The conductivity of the prepared hydrogels was tested by a RTS-8 four-probe tester (Guangzhou Four Probe Technology Co., Ltd.).

### 2.3 Experimental Methods

#### 2.3.1 Preparation of Ag NPs

A 97-ml silver nitrate solution (0.1 × 10^−3^ M) was prepared and stirred vigorously at room temperature. Then 1 ml of sodium citrate solution (180 × 10^−3^ M), 1 ml of PVP aqueous solution (4.2 × 10^−3^ M), and 240 μL of H_2_O_2_ solution (30 wt%) were introduced in sequence. Finally, 800 μL of NaBH_4_ solution (100 × 10^−3^ M) was added. After a few minutes, the color of the mixture solution changed rapidly from transparent to dark blue, and stirring was continued for more than 5 h. Afterward, a stable 0.1 × 10^−3^ M antibacterial Ag NPs solution was obtained and labeled for later use. The TEM image of the prepared Ag NPs is shown in Supplementary Figure S1.

#### 2.3.2 Preparation of CPH

First, 0.25 g of PVA and 0.075 g of gelatin were placed into 4.675 ml of ultrapure water. The solution was stirred vigorously at 90°C until the raw materials were completely dissolved. Then, the obtained mixed solution was transferred to a magnetic stirrer at room temperature, and 1 ml of PA solution, 600 μL of AN, and 1 ml of APS solution were added to the mixture as the initiator. The solution color changed from transparent to dark green immediately. Then, the sol was placed in the refrigerator at −20°C overnight to prepare the gel. The hydrogel was removed the next day and soaked in ultrapure water to remove impurities. The ultrapure water was replaced every 8 h, and this process was repeated six times. Thus, the conductive CPH material was obtained.

#### 2.3.3 Preparation of Ag NPs/CPH

The prepared CPH material was immersed in the Ag NPs solution and removed after 24 h to prepare the Ag NPs/CPH. The Ag NPs/CPH solution was placed in a glass container, sealed with a parafilm, and stored at 4°C in the dark.

#### 2.3.4 Bacteriostatic Properties of Ag NPs/CPH

Diluted bacterial solutions of two of the most common bacteria in wound infections, *E. coli* and *S. aureus*, were evenly spread on an agar plate. Then, 400 μL of CPH hydrogel sheets were loaded with Ag NPs at immersion concentrations of 0 M (denoted as CPH), 0.1 × 10^−3^ M (denoted as 1 Ag), 0.2 × 10^−3^ M (denoted as 2 Ag), 0.3 × 10^−3^ M (denoted as 3 Ag), and 0.4 × 10^−3^ M (denoted as 4 Ag) were placed on the plate and refrigerated for 1 h at 4°C. The plates were then placed upside down and cultured at 37°C for 12 h, and the size of the bacteriostatic zone was measured in each plate.

To further investigate the diffusion and antibacterial effects of the Ag NPs in the prepared hydrogels, a Luria-Bertani culture medium (denoted as lb) and diluted bacterial solutions of *E. coli* and *S. aureus* were used as the control groups (denoted as *E. coli* and *S. aureus*). In addition, CPH hydrogel sheets loaded with Ag NPs (1 Ag, 2 Ag, 3 Ag, and 4 Ag) were added to each test tube with equally diluted bacterial culture media. The OD values of the above media were measured at 0, 2, 6, 12, 24, and 48 h (timing started when the bacteria began gradually growing in the liquid medium).

#### 2.3.5 Cytotoxicity Test of Ag NPs/CPH

First, a 5 g sample of Ag NPs/CPH was placed in a centrifuge tube containing 30 ml of PBS, which was then soaked in an incubator at 37°C for 24 h. After removing the Ag NPs/CPH material, the concentration of the soaking solution in the tube was measured. Next, ultrapure water was introduced to obtain a series of gradient Ag NPs/CPH soaking solution concentrations (10, 20, 30, 50, 60, 80, 100, and 150 μg/ml). The CCK-8 method was used to investigate the toxicity of the antibacterial hydrogel soaking liquid in immortalized human epidermal cells (HaCat), human normal liver cells (LO2), and human embryonic kidney cells (293T) under different concentrations.

#### 2.3.6 Evolution of Ag NPs/CPH for Infected Wound Healing

All the animals were acclimated under standard laboratory conditions (ventilated room, 25°C ± 1°C, 60 ± 5% humidity, 12-h light/dark cycle) and had free access to standard water and food (202100035933). All procedures were conducted in accordance with the Guiding Principles in the Care and Use of Animals (China) and approved by Animal Experimental Ethical Inspection Form of Changchun University of Chinese Medicine (2021062).

Male ICR mice were divided into gauze (control), CPH, and Ag NPs/CPH groups. A wound model was made on the back of each animal and the size of the wound was measured. Equal concentrations of *S. aureus* bacteria fluid were applied to each would, causing infection. After 24 h of normal activity and without any additional treatment, the wounds became red and swollen, with a small amount of surrounding exudate. The different patches (control, CPH, Ag NPs/CPH) were applied to the wounds of the ICR mice, according to the different groups. All areas of each wound were measured and photographed after 1, 2, 4, 7, and 14 days. Then, the mice were euthanized and the skin tissue near the wound was stored in paraformaldehyde in a refrigerator at 4°C. After 14 days, tissue sections were embedded and uniformly stained, and inflammation and tissue repair were individually observed under a microscope at 400x magnification.

## 3 Results and Discussion

**FIGURE 1:**
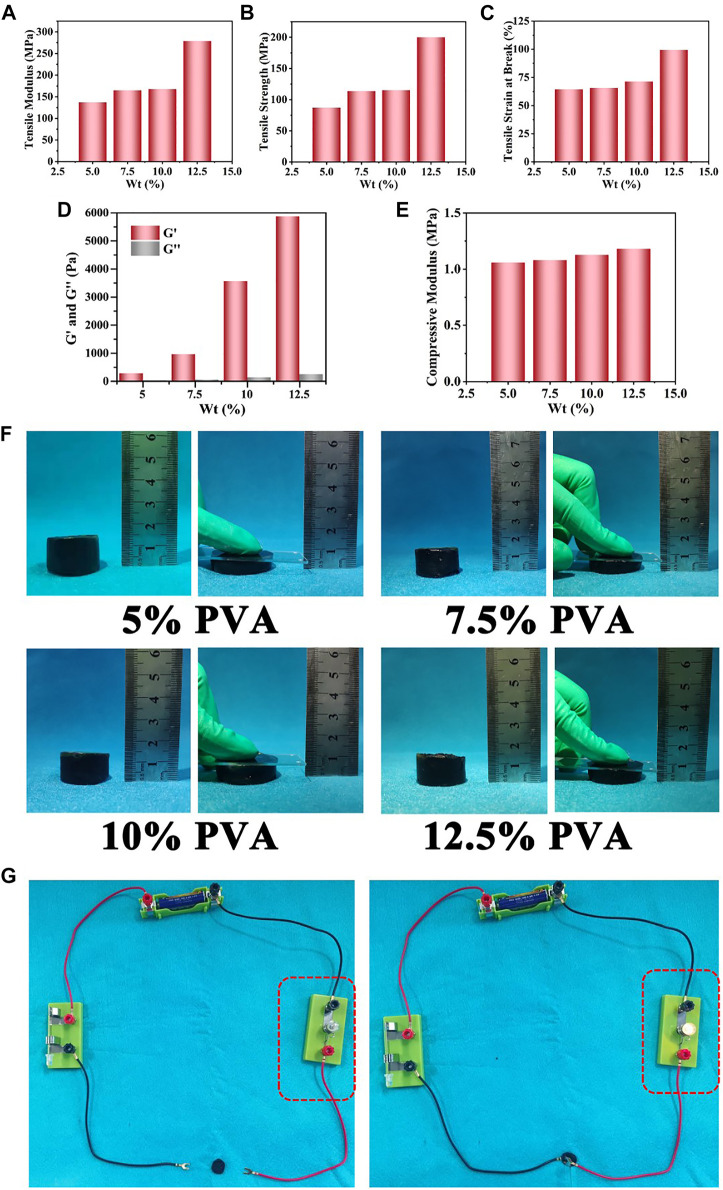
The tensile and compression performance tests of the Ag NPs/CPH material prepared with different amounts of reactive PVA content (5, 7.5, 10, and 12.5 wt%): **(A)** tensile modulus of elasticity. **(B)** Tensile strength. **(C)** Tensile strain at breakage. **(D)** The storage (G′) and loss (G″) modulus. **(E)** Compressive modulus. **(F)** The photos of compressive deformation effect of prepared PVP@Ag NPs/CPH with different PVA concentrations under a certain pressure. **(G)** Conductive Ag NPs/CPH (with AN reactive volume of 600 μL) connected to a circuit, which made the bulb glow.

Because PVA was used as the main matrix material, the effects of PVA content on the mechanical properties of the prepared Ag NPs/CPH material were investigated. Within a certain reactive PVA hydrogel concentration range (≈2.5—10 wt%), the Ag NPs/CPH material was prepared successfully. As shown in Figures 1A–C, and Supplementary Figure S2B, the increasing reactive content of PVA (5, 7.5, 10, and 12.5 wt%), the tensile modulus, tensile strength, and the elongation at break steadily increased. At the same time, the corresponding storage modulus (G′) of the above hydrogels increased significantly, implying the increase of the hydrogel rigidity (Figure 1D and Supplementary Figure S3). Supplementary Figure S4 shows the strain amplitude scanning of hydrogels with different PVA content. In the low strain area (<10%), the G′ and G″ of each hydrogel were relatively constant. As the strain increased, the G′ and G″ curves gradually intersected, and the critical point gradually moved forward as the PVA reaction concentration increased. It indicated the prepared hydrogel was becoming harder. When the strain was further increased, due to the breakage and collapse of the macromolecular chain of the hydrogel network, all the G′ and G″ values were both significantly reduced. Similarly, the compressive modulus of the hydrogels increased as the PVA reaction concentration increased (Figure 1E and Supplementary Figure S2A). However, the increase in the compressive modulus was relatively low. As shown in Figure 1F, when pressure was applied to the surface of the hydrogels prepared with different reactive concentrations of PVA, the degree of compression was similar. After the pressure was removed, the hydrogel returned to its original state, with few macroscopic changes. The dynamic storage modulus (E′) and loss modulus (E″) of the hydrogel with different PVA concentrations were also assessed using a dynamic thermomechanical analyzer (Supplementary Figure S5).

These results showed that the lower the PVA content, the softer the hydrogel. All of the prepared hydrogels had a certain strength and modulus, which ensured that the materials were not easily damaged during processing and use. During the experiment, when the PVA content was 2.5% or less, the prepared hydrogel was easily broken, as there was less PVA matrix content, which was not conducive to hydrogel formation. When PVA content was 10% and higher, curing occurred too quickly. This caused the system to become non-homogeneous, and the hydrogel became too rigid and large for molding. Softer hydrogels adhere more easily to the skin, which reduces the amount of squeezing and irritation to the wounds during use. As a result, a PVA concentration of 5 wt% was selected to prepare the CPH.

To achieve a better material for use in bioelectric sensing, hydrogels with different reactive volumes (400, 600, and 800 μL) of aniline were prepared. When the aniline reactive volume was 200 μL, the hydrogel was not conductive. However, when the reaction volume of aniline increased to 1,000 μL, the hydrogel exhibited poor moldability and was easily broken. To test conductivity, a circuit was connected to a 1.5-V power supply, and two wires were pressed onto the prepared hydrogel surface with an insulating glass plate. As shown in Figure 1G, when the reactive content of PANI was 400, 600 and 800 μL, the bulb emitted a bright light. Their corresponding conductivity were tested by a RTS-8 four-probe tester to be 0.032, 0.054 and 0.069 S/cm, respectively. It confirmed that all the prepared hydrogels had good electrical conductivity and could be used as conductive skin-adhesive medical dressings. Supplementary Figure S6 shows a diagram of the dielectric constants of the prepared hydrogels obtained using a dielectric impedance meter with different amounts of reactive AN (400, 600, and 800 μL).

**FIGURE 2:**
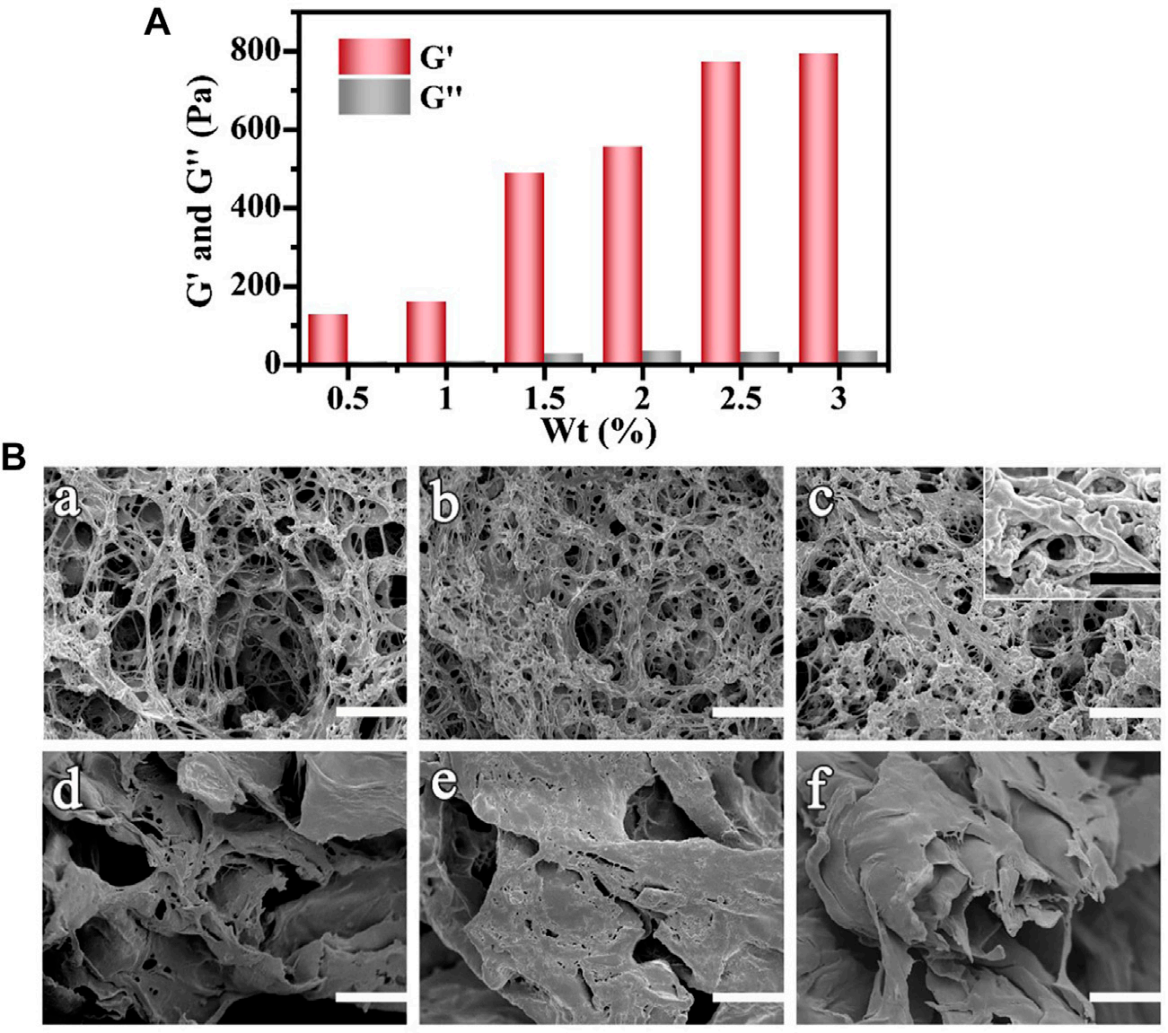
**(A)** G′ and G″ vs. the concentration of gelatin increases from 0.5 to 3 wt%. **(B)** SEM images of Ag NPs/CPH material with different amounts of gelatin content (a) 0.5 wt%, (b) 1 wt%, (c) 1.5 wt%, (d) 2 wt%, (e) 2.5 wt%, and (f) 3 wt%.

As the other main component of CPH, gelatin, which is composed of multiple triple helixes, has a dense structure. Excessive gelatin reaction content may result in a hydrogel that is too dense and has low internal porosity. This would inhibit the high loading and gradual release of the Ag NPs. Thus, based on the reaction amount of the host PVA material and the conductive AN material, the effects of water-absorption and gelatin content on the prepared CPH structure were investigated. In Figure 2A, Supplementary Figure S7, and Supplementary Figure S8, as the gelatin reactive concentration increased from 0.5 wt% to 3 wt%, the G′ increased. Specifically, the rigidity of the hydrogel increased. Figure 2B shows the SEM images depicting the internal topography of the corresponding hydrogels. When the gelatin content was 0.5 wt% (Figure 2Ba), the prepared hydrogel had a loose and microporous interior structure with poor mechanical properties, as well as low porosity and specific surface area. As the reaction content of gelatin gradually increased (1 and 1.5 wt% in Figure 2Bb and Figure 2Bc, respectively), the hydrogel interior became gradually denser, and the pore diameter became smaller with a higher specific surface area. Under higher magnification (insert in Figure 2Bc), the typical coral-like and dendritic 3D network structure of PANI was observed.^28^ As the gelatin content continued to increase (2, 2.5, and 3 wt% in Figure 2Bd, Figure 2Be, and Figure 2Bf, respectively), the hydrogel interior became very dense with fewer pores for loading Ag NPs or liquid preservation. It corresponds to the test results of G′, shown in Figure 2A. Thus, a gelatin reaction amount of 1.5 wt% was selected to achieve a CPH material with excellent porosity, specific surface area, and water absorption.

According to the above analysis results and previous reports, the material containing 5 wt% of PVA concentration, 1.5 wt% of gelatin content, 600 μL of AN reactive volume, 600 μL of PA reactive volume was chosen for CPH preparation (Gao et al., 2015; Butylina et al., 2020). After freezing and thawing for 10 h, the hydrogel solution changed from a liquid sol to a stable gel. The gel did not flow when placed at an angle, as shown in Supplementary Figure S9A. Of note, the sol was poured into molds of various shapes before freezing, and the material was removed from the mold after undergoing freezing and thawing. Hence, CPH hydrogels with desired sizes and shapes were successfully obtained (Supplementary Figure S9B). When removed from the molds, the prepared hydrogels exhibited good elasticity and maintained good mechanical properties after being subjected to certain external forces, such as stretching, compression, or shearing treatment. These properties are beneficial for storage, transport, or tailoring the material for use in practical applications.

**FIGURE 3:**
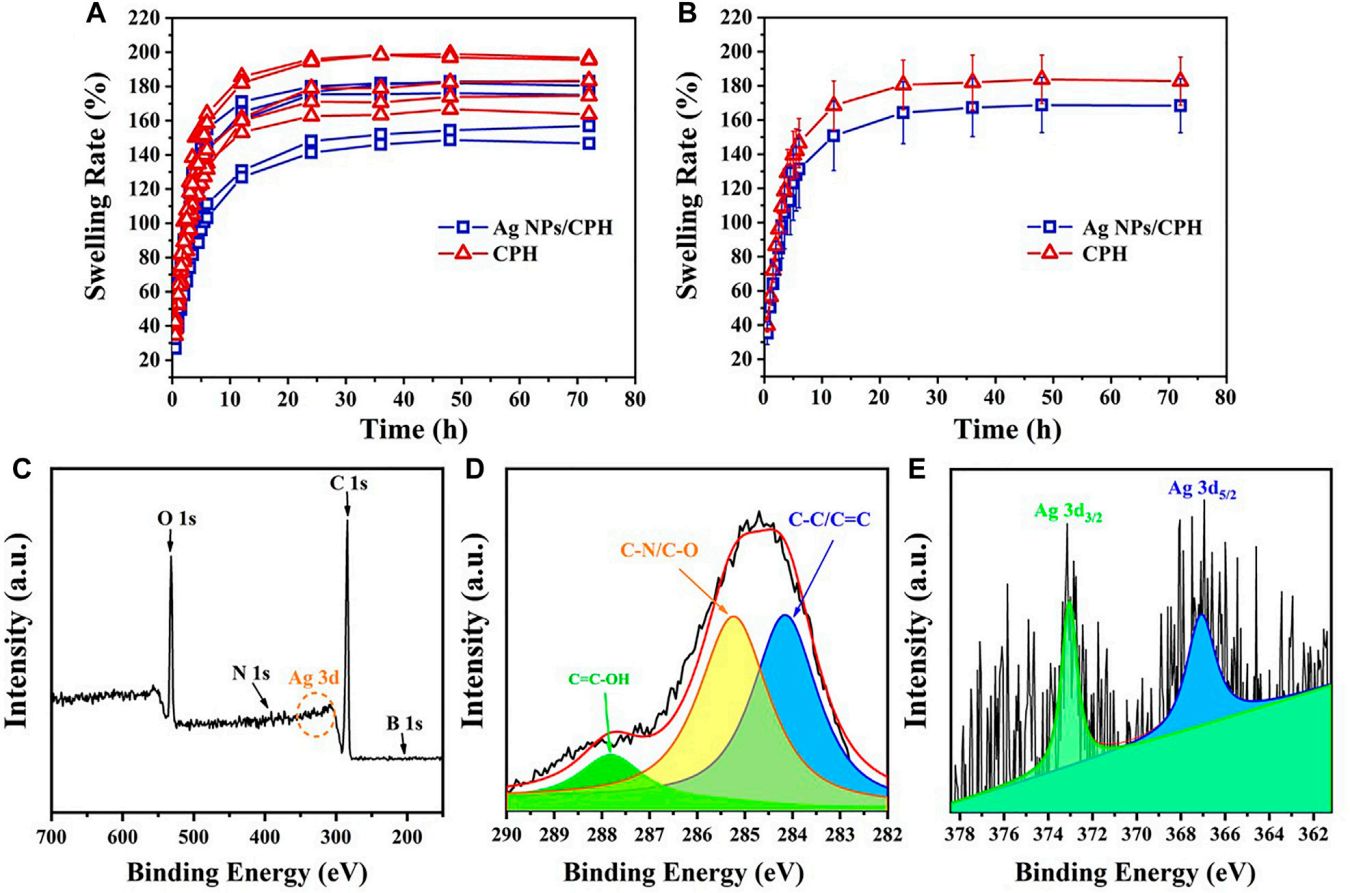
**(A)** Ten volume swelling curves for the five CPH and Ag NPs/CPH sample groups from 0 to 72 h. **(B)** The statistical result curve of **(A)**. **(C)** Bull scanning XPS spectrum of Ag NPs/CPH. **(D,E)** show the high-resolution XPS spectra of C 1s and Ag 3d, respectively.

Hydrogel swelling plays an important role in wound healing. An ideal inflammatory wound dressing should absorb large amounts of inflammatory wound exudate, maintain the wound in a moist environment, accelerate granulation and blood vessel formation, and promote the self-dissolution of necrotic tissue. In our work, the five prepared CPH and Ag NPs/CPH samples sets were dried at 40°C for 24 h to study their swelling properties. Figure 3A shows the volume swelling curve of the 10 samples under excessive moisture conditions from 0 to 72 h. As shown in the Figure 3B, the dried sample continued to rapidly absorb water with swelling rated to be 100% and expand within ≈6 h. Between 6 and 12 h, the water absorption rate (140–160%) of the samples slowed down, with a relatively fast water absorption rate. After 12 h, the swelling rate (160–180%) of the hydrogels plateaued over an extended period of time (≈72 h). This suggested that the prepared hydrogels could continuously absorb water and swell for a relatively long time. In addition, the material could store the absorbed liquid inside the internal pores longer, to be used at a later time. Thus, the material had good water absorption and stability characteristics. We also inferred that when used as a wound dressing, the loaded Ag NPs in the Ag NPs/CPH material could exchange with the wound exudate along a concentration gradient. Thus, the Ag NPs could continuously act on the wound surface, effectively inhibiting bacteria. Furthermore, the wound exudate did not exude again after absorption into the hydrogel, avoiding secondary infection to the wound.

To determine the composition of the prepared hydrogel, a freeze-dried hydrogel sample was analyzed by FTIR, as shown in Supplementary Figure S10. In the FTIR spectrum of Ag NPs/CPH (red line), the peak at 3,226 cm^−1^ was derived from -OH from PVA and gelatin, and the bands at 1,648 cm^−1^ and 1,656 cm^−1^ were derived from C-O in both PVA and gelatin. In the FTIR spectrum of AN, the position peak at 1,558 cm^−1^ corresponded to the stretching vibration of the benzene ring, and the position peak at 3,425 cm^−1^ came from -NH_2_. However, the position peak of AN at 3,425 cm^−1^ disappeared, while a new position peak at 1,288 cm^−1^ appeared. This was a characteristic peak of the tertiary amine functional group. In addition, a peak at 883 cm^−1^ appeared, which originated from double substitution on the benzene ring. For PA, the position peak at 2,360 cm^−1^ was derived from O=P-OH···O, which did not appear in the FTIR spectrum of Ag NPs/CPH. By contrast, the position peaks of 1,564 cm^−1^ (-NH), 1,656 cm^−1^ (-C=O), and 1,421 cm^−1^ (-CN) in the amide group appeared, which also indicated cross-linking between PA and PANI during Ag NPs/CPH synthesis.

Figure 3C shows the full XPS spectrum of the prepared Ag NPs/CPH. The binding energies of C1s were 287.7, 285.3 and 284.7 eV, indicating -COOH, C=O/C-N, and C=C/C-C functional groups (Figure 3D). The binding energies of 373.2 and 367.1 eV corresponded to Ag 3d, which confirmed that the Ag NPs were successfully loaded into the prepared hydrogel (Figure 3E). As observed in the figure, the obtained Ag 3d XPS peak was weak. This was presumably because the Ag NPs in the sample were mainly present in the internal pores of the hydrogel, and were less attached to the surface of the hydrogel in the dry state.

**FIGURE 4:**
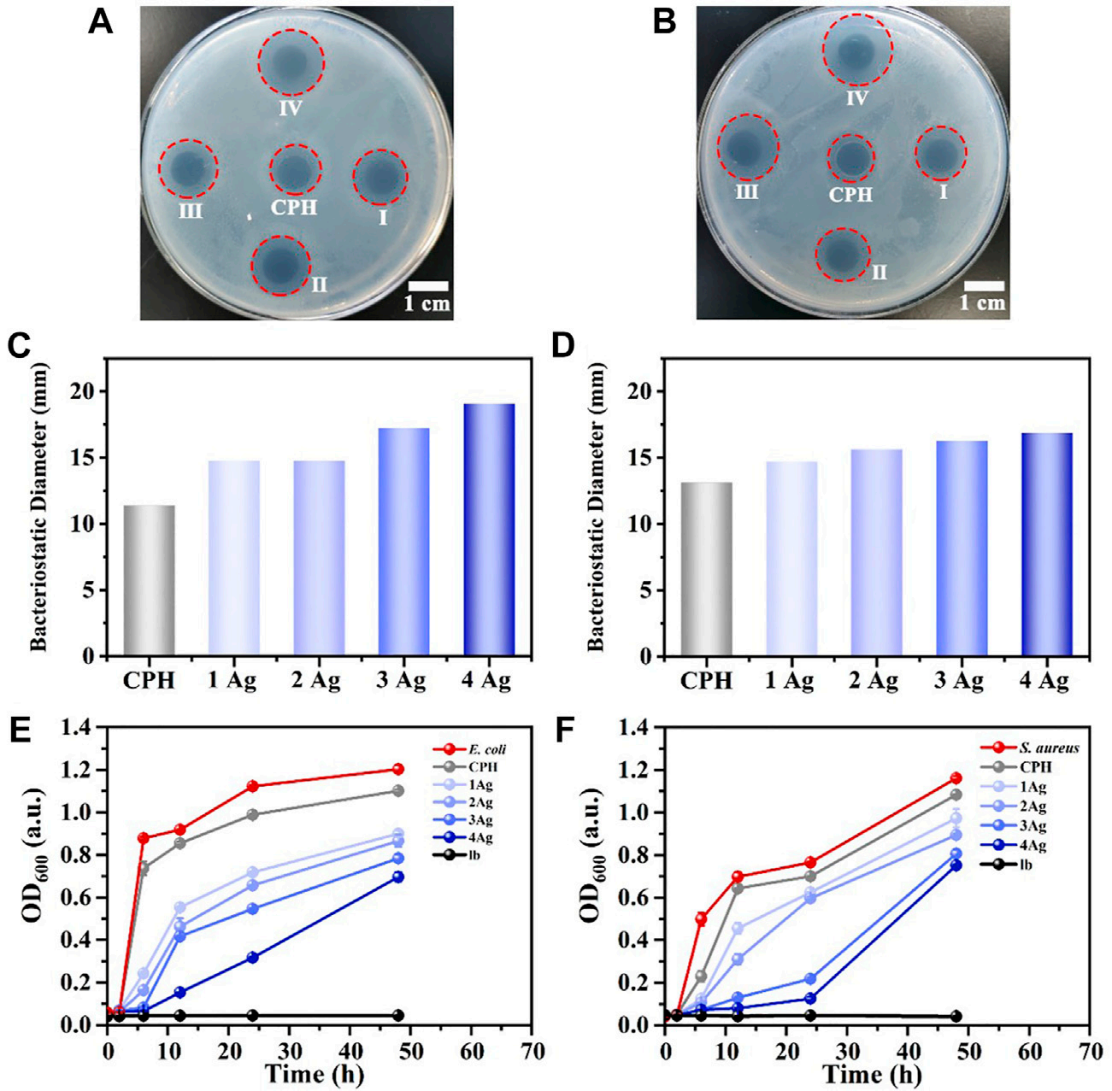
**(A,B)** show the photos of the antibacterial plate experiment (*E. coli* and *S. aureus*) for Ag NPs/CPH. In the photos, I denotes the 1 Ag group, II denotes the 2 Ag group, III denotes the 3 Ag group, and Ⅳ denotes the 4 Ag group. **(C,D)** show the calculated diameter of the inhibition zone of the corresponding antibacterial plate experiment. **(E)** OD value diagram of the lb medium, CPH, *E. coli* solution, and *E. coli* cocultures with Ag NPs/CPH at different concentrations of Ag NPs (1 Ag, 2 Ag, 3 Ag, and 4 Ag) from 0 to 48 h. **(F)** OD value diagram of the lb medium, CPH, *S. aureus* solution, and *S. aureus* cocultures with Ag NPs/CPH at different concentration of Ag NPs (1 Ag, 2 Ag, 3 Ag, and 4 Ag) from 0 to 48 h.

To determine the antibacterial properties of the prepared Ag NPs/CPH, hydrogel sheets loaded with Ag NPs at immersion concentrations denoted by CPH, 1 Ag, 2 Ag, 3 Ag, and 4 Ag in the antibacterial plate and co-cultivation with bacteria (*E. coli* and *S. aureus*) experiments were assessed. Figures 4A,B depicts the Ag NPs/CPH samples immersed in different concentrations of Ag NPs aqueous solutions, showing that they released the antibacterial Ag NPs, effectively inhibiting the reproduction of *E. coli* and *S. aureus* bacteria. The calculated size of the inhibition zone (Figures 4C,D) on the plate increased with increased Ag NPs solution concentration. In addition, the CPH without immersion in the Ag NPs solution displayed a bacteriostatic zone, possibly due to the antibacterial properties of PANI. However, due to the lack of Ag NPs, the inhibition zone area was significantly smaller compared to Ag NPs/CPH.

Figure 4E (*E. coli*) and F (*S. aureus*) shows the OD value diagram of the lb medium, CPH, bacterial solution, and bacterial cocultures with Ag NPs/CPH at different concentration of Ag NPs (1 Ag, 2 Ag, 3 Ag, and 4 Ag) from 0—48 h under identical culture conditions. In Figure 4E, the OD value of the lb medium hardly changed over time as it contained considerably fewer bacteria. The OD values in the *E. coli* and CPH groups showed nearly identical trends in bacterial growth. From 2 to 6 h, the OD values of the two groups rose rapidly, indicating the logarithmic growth phase of bacterial growth, and from 6 to 24 h, both OD values rose slowly, indicating the stable period of bacterial growth. After 24 h, the OD values were unchanged, indicating the slow period of bacterial growth. Because CPH had certain antibacterial properties, its OD value was lower than that of the *E. coli* group. Although all hydrogel groups loaded with Ag NPs exhibited similar bacterial growth curves to the *E. coli* and CPH groups, the OD values of the 1 Ag, 2 Ag, 3 Ag, and 4 Ag groups showed a significant downward trend. In addition, as Ag NPs loading increased, the OD values of the cocultured bacterial solution noticeably decreased. Among these, the 4 Ag group displayed the best antibacterial effect. These results were consistent with the results observed in Figures 4A,C.

Ag NPs/CPH inhibited *S. aureus* with similar results, as shown in Figure 4F. It is worth mentioning that for *S. aureus*, the inhibitory effects of the 3 Ag and 4 Ag groups were the best. The above experimental results also implied that the prepared hydrogels had an abundant number of pores, which could be loaded to continuously release Ag NPs and achieve good antibacterial properties. In addition, in practical applications, Ag NPs impregnation can be adjusted according to the type of bacteria in the wound, to achieve the best inhibitory effect. Additionally, positive and negative controls about the antimicrobial activity of the nanocomposite hydrogel have been discussion in Supplementary Figure S11.

**FIGURE 5:**
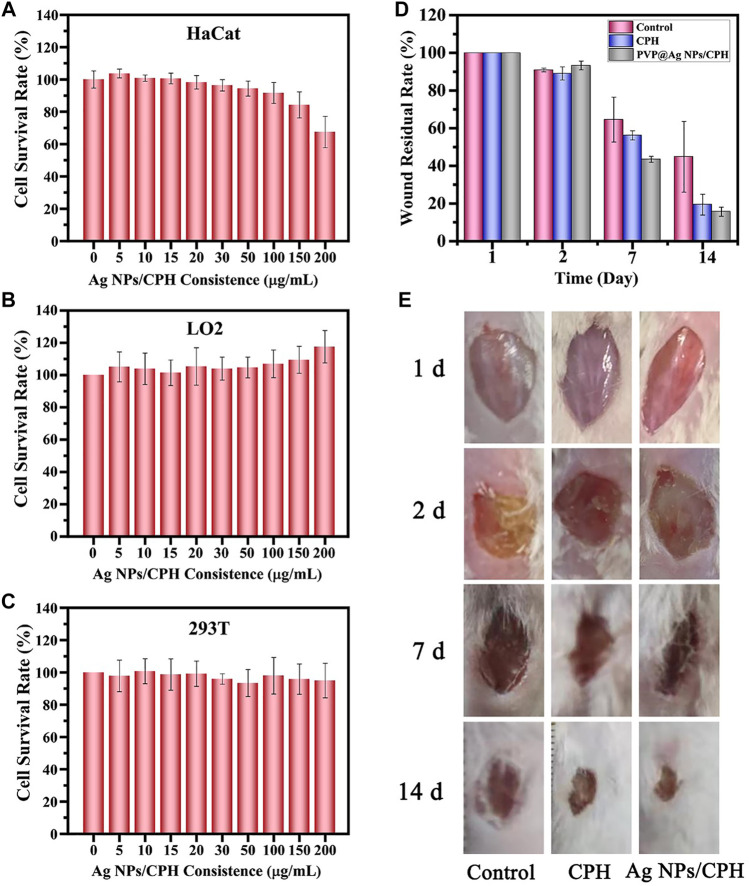
**(A)** HaCat, **(B)** LO2, and **(C)** 293T cell survival rates measured using the CCK-8 method in pure PBS with hydrogel soaking solution (10, 20, 30, 50, 60, 80, 100, 150, and 200 μg/ml). **(D)** Corresponding remaining areas of the wound in the three different treatment groups at 1, 2, 7, and 14 days. **(E)** Photos of mouse wounds treated with PBS as the control, CPH, and Ag NPs/CPH group.

Some literature studies have reported that large doses of silver nanoparticles may have inhibitory effects on some tissue cells (Gao et al., 2017). Based on the concentration of Ag NPs in literature (Tak et al., 2015), their cytotoxicity was investigated through the toxicity experiment of the hydrogel soaking solution on three normal cells. HaCat cells are immortalized human keratinocytes that have undergone self-transformation. Their biological characteristics are similar to normal keratinocytes. Thus, they are suitable for various cell studies to investigate the skin toxicity of different materials. The toxicity tests of Ag NPs/CPH on HaCat cells were performed via the CCK-8 method, to observe their effects on human skin. HaCat cells were co-cultured with a control group consisting of pure PBS and a soaking solution of Ag NPs/CPH hydrogels (5, 10, 15, 20, 30, 50, 100, 150, and 200 μg/ml) for 48 h. As shown in Figure 5A, compared to the control group (0 μg/ml), the low-concentration (5, 10, and 15 μg/ml) groups showed minimal inhibitory effects and even had a tendency to proliferate the cells. This showed that for ordinary wounds, small amounts of Ag NPs/CPH were non-toxic to skin cells, and possibly promote the growth of skin cells and accelerate skin healing. Under the same conditions, after co-cultivation with the hydrogel soaking solution at medium concentrations (20, 30, 50, and 100 μg/ml), HaCat cells were slightly inhibited (<10%). When the soaking solution concentration increased to 150 μg/ml, the HaCat cells were inhibited to a lesser extent (<20%), indicating low cytotoxicity of Ag NPs/CPH on HaCat cells. Thus, when a large amount of Ag NPs/CPH was used for severe wounds (such as inflammation and purulent), it would not cause adverse effects on wound healing. Therefore, we inferred that the prepared hydrogel loaded with Ag NPs in this study exhibited good antibacterial properties and was non-toxic. To further examine the *in vivo* safety of Ag NPs/CPH, CCK-8 experiments of LO2 and 293T cells were carried with different concentration (5, 10, 15, 20, 30, 50, 100, 150, and 200 μg/ml) of the Ag NPs/CPH hydrogel soaking solution. The experimental results (Figures 5B,C) showed that each concentration of Ag NPs/CPH soaking solution had no significant inhibitory effect on LO2 and 293T cells, with even slight proliferation.

The effects of Ag NPs/CPH dressing on infected wounds in mice are shown in Figure 5E. The gauze dressing was used as the control group, and the CPH and Ag NPs/CPH dressing groups were the experimental groups. On the first day, the wounds (diameter ∼1 cm) of the three groups of mice were all clear, with no bleeding or exudation. After evenly applying the *S. aureus* bacterial solution, there were no obvious signs of infection within a short period of time. 20 h later on the second day, the wounds of the three groups of mice were infected and purulent, with an obvious peculiar smell. Thus, a severe infection model was successfully established. The mice in each group were given corresponding dressings (diameter ≈1.2 cm) to treat the wounds. On the seventh day, adhesion of the gauze (Supplementary Figure S12) and thick scabs near the wounds were observed in the mouse control group. When the dressing was changed, the mice showed signs of pain. However, the wounds of the mice in the CPH and Ag NPs/CPH groups showed improvement. There was no adhesion when the dressing was changed, the mice struggled less, and no secondary wound injuries were observed. The residual areas of the wounds of the mice in the Ag NPs/CPH group were the smallest. At 14 days, the mice in the control group still showed larger residual wound areas, while the wounds of the mice in the CPH and Ag NPs/CPH groups were almost covered by new hair. Of note, the mice in the Ag NPs/CPH group showed the smallest residual wound area. This proved that Ag NPs/CPH had obvious advantages such as antibacterial, anti-infection, and healing properties, compared to traditional dressings (such as gauze) when treating severely infected wounds.

Figure 5D shows the statistical calculation diagram of the remaining wound areas of the mice in each group, between 2 and 14 days. On the second day, 20 h after severe infection with *S. aureus*, the wound areas of the three groups of mice all reduced to about 90% of their original size. On the seventh day, there were significant differences in the wound areas in the three groups. The wound area of the control group was still greater than 60%, and the wound area of the CPH group was slightly less than 60%. The wound area of the Ag NPs/CPH group was close to 40%, with the best recovery effect. At 14 days, the wound area of the control group was still close to 40%, while the CPH group was ∼20%. Notably, the wounds of the mice in the Ag NPs/CPH group were nearly healed, with a wound area close to 10%. In brief, the Ag NPs/CPH group showed the best healing effect, and the CPH group performed better than the control group, which was consistent with the results shown in Figure 5B.

**FIGURE 6:**
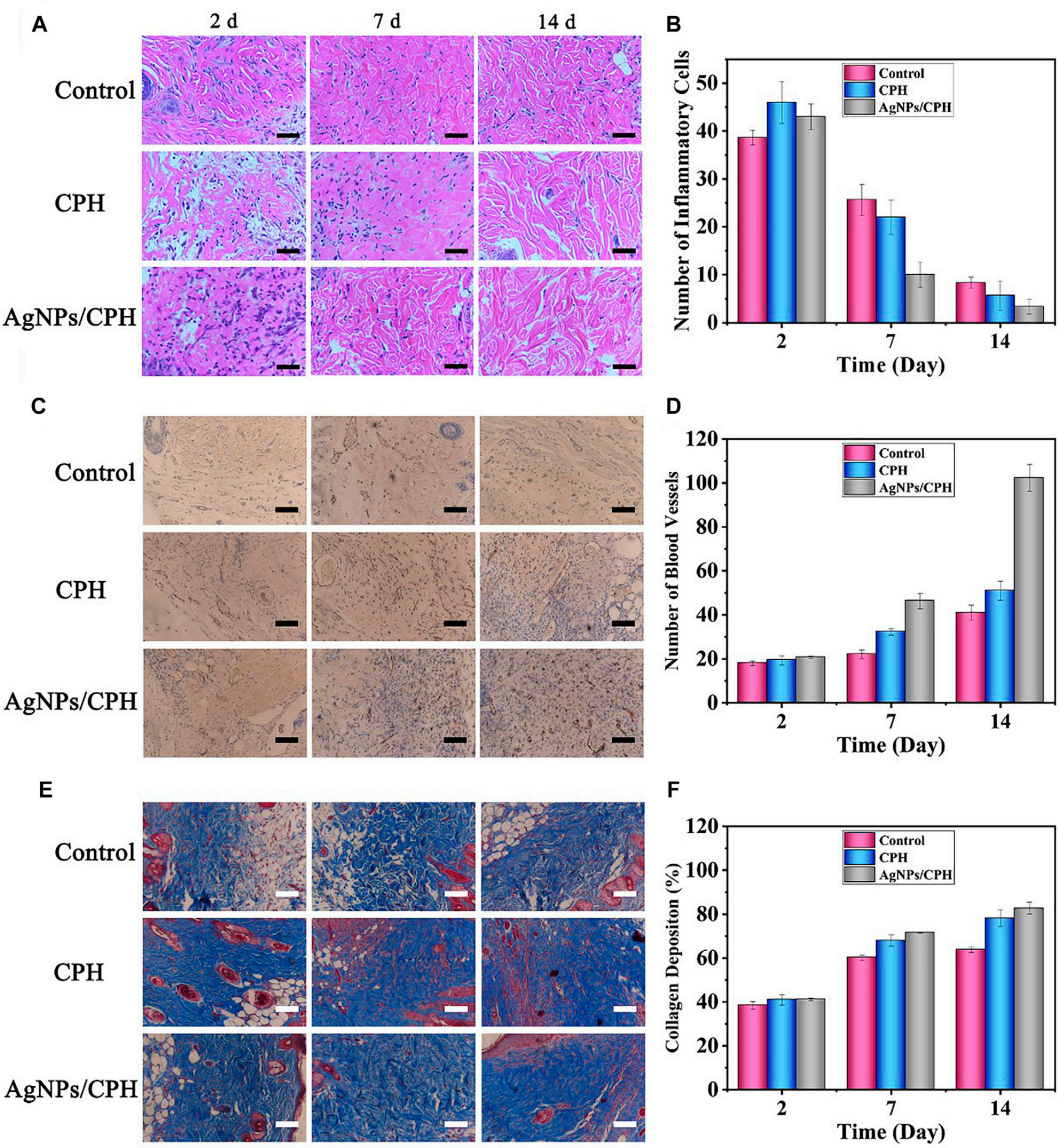
**(A)** H&E-stained sections of skin tissue near the wound of mice treated with gauze (control group), CPH, and Ag NPs/CPH after 2, 7, and 14 days (scale: 50 μm). **(B)** Corresponding count of inflammatory cells. **(C)** CD31-stained sections of skin tissue near the wound of mice treated with gauze (control group), CPH, and Ag NPs/CPH after 2, 7, and 14 days (scale: 100 μm). **(D)** Corresponding count of blood vessels in Figure 6C. **(E)** Masson-stained sections of skin tissue near the wound of mice treated with gauze (control group), CPH, and Ag NPs/CPH after 2, 7, and 14 days (scale: 100 μm). **(F)** Corresponding percentage of collagen deposition in Figure 6E.

Figure 6A shows the H&E-stained section of the skin tissue obtained near the wound, as observed under a microscope after 2, 7, and 14 days. Figure 6B shows the corresponding count of inflammatory cells. When the severely infected wound model of the three mice groups was established at 2 days, the number of inflammatory cells in the three groups was very high. At 7 days, after treatment with different dressings, the number of inflammatory cells in the control group was still higher in the stained sections, and the number of inflammatory cells in the CPH and Ag NPs/CPH groups was lower than in the control group. In particular, the number of inflammatory cells in the Ag NPs/CPH group was greatly reduced compared with the other two groups. This indicated that the Ag NPs/CPH dressing performed better in controlling the inflammation of the wound. After 14 days, the inflammatory period of the three mice groups had finished. Some inflammatory cells in the control group were still observed, while the number of inflammatory cells in the Ag NPs/CPH group was less than half that of the control group. Inflammation in the mice in the Ag NPs/CPH group was almost imperceptible.

Figure 6C shows the CD31-stained section of the skin tissue obtained near the wound, after 2, 7, and 14 days. The statistical count of blood vessels is shown in Figure 6D. At 2 days, due to the skin defects, the number of blood vessels in each group was small, under 20. At 7 days, sampling and counting performed near the newly added granulation tissue showed the three groups had differences in the number of blood vessels: there were slightly more blood vessels in the control group. Both the CPH and Ag NPs/CPH groups had a clear increase in the number of blood vessels, especially the Ag NPs/CPH group, which reached ≈40. At 14 days, the number of blood vessels near the wound in each group increased considerably. The most obvious of increase was in the Ag NPs/CPH group, where the number of blood vessels in the slice reached 100.

Figures 6E,F display the Masson-stained section of the skin tissue obtained near the wound after 2, 7, and 14 days. The deposition rate of collagen in each group increased over time. CPH and Ag NPs/CPH groups had faster increases in collagen content than the control group, indicating better wound healing. The results shown in Figure 6 are consistent with the conclusion shown in Figure 5E. This further indicated that the hydrogel patch prepared in this work has an excellent promotive effect on the healing of severe wounds.

## 4 Conclusion

Biocompatible PVA and gelatin were used as main matrix materials, compounded with a PA cross-linked PANI network, and loaded with Ag NPs through dipping to synthesize a novel medical Ag NPs/CPH hydrogel dressing. Through tensile, SEM, and electrical property studies, the optimal reactive concentration of PVA, gelatin, and AN were determined. After fully soaking in an aqueous Ag NPs solution, a skin-adaptable conductive medical Ag NPs/CPH hydrogel dressing was obtained. Furthermore, FTIR and XPS analyses confirmed the components of the prepared hydrogels. The biological toxicity, antibacterial, and infected wound treatment properties were investigated using a mouse model. The results showed that the obtained Ag NPs/CPH material had good mechanical properties, abundant internal pores, good electrical conductivity, effective antibacterial properties, low biological toxicity, and good healing ability for infected wounds.

Of note, in the biological experiments, we designed an innovative, severely infected animal wound model, with wounds that were infected with bacteria for a long time (more than 20 h). Through a series of biological experiments, the prepared antibacterial hydrogel dressings showed potential for treating infected and inflammatory human wounds. Therefore, this work established a new research idea and reference basis for the future design and application of composite medical hydrogel dressings.

## Data Availability

The original contributions presented in the study are included in the article/Supplementary Material, further inquiries can be directed to the corresponding authors.
