# Supplementary material for: A Novel Conductive Antibacterial Nanocomposite Hydrogel Dressing for Healing of Severely Infected Wounds
Source: Front Chem. 2021 Nov 24;9:787886. doi: 10.3389/fchem.2021.787886 (PMC8652251; doi:10.3389/fchem.2021.787886)
Supplement: Supplementary file 1 [file DataSheet1.docx]

# Supporting Information

**A Novel Conductive Antibacterial Nanocomposite Hydrogel Dressing for Healing of Severely Infected Wounds**

Lizhi Xiao^a, #^, Fang Hui^a, #^, Tenghui Tian^a^, Ruyue Yan^a^, Jingwei Xin^b^, Xinyu Zhao^a^, Yingnan Jiang^a, *^, Zhe Zhang^a, *^, Yulan Kuang^a^, Na Li^c^, Yu Zhao^a, *^ and Quan Lin^b^

^#^These authors contributed to the work equally and should be regarded as co-first author.

^a^ Jilin Ginseng Academy; Hospital of Affiliated Changchun University of Chinese Medicine, Changchun University of Chinese Medicine, Changchun, 130117, P. R. China;

^b^ State Key Laboratory of Supramolecular Structure and Materials, College of Chemistry; Chinese-Japan Union Hospital of Jilin University, Jilin University, Changchun, 130012, P. R. China;

^c^ Key Laboratory of Songliao Aquatic Environment of Ministry of Education, Jilin Jianzhu University, Changchun, 130118, P. R. China.

**Corresponding Authors**

*Y. J.: e-mail, jiangyn@ccucm.edu.cn

*Y. Z.: e-mail, [cnzhaoyu1972@126.com](mailto:cnzhaoyu1972@126.com)

*Z. Z.: e-mail, zhangzhe@ccucm.edu.cn


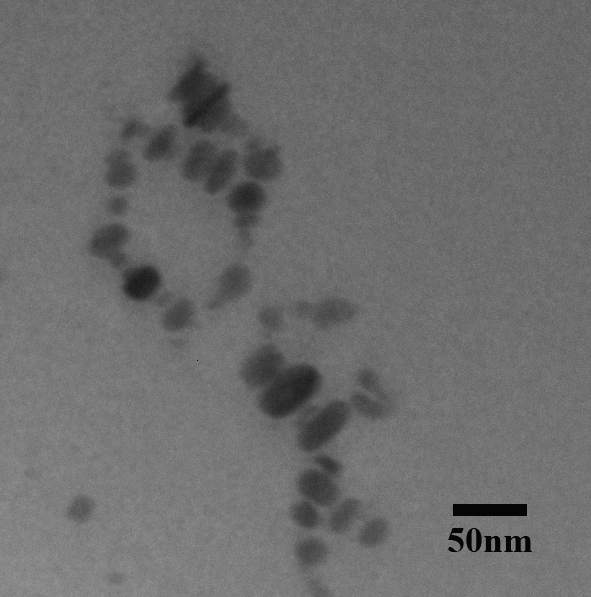


**Fig. S1** TEM image of the prepared Ag NPs.


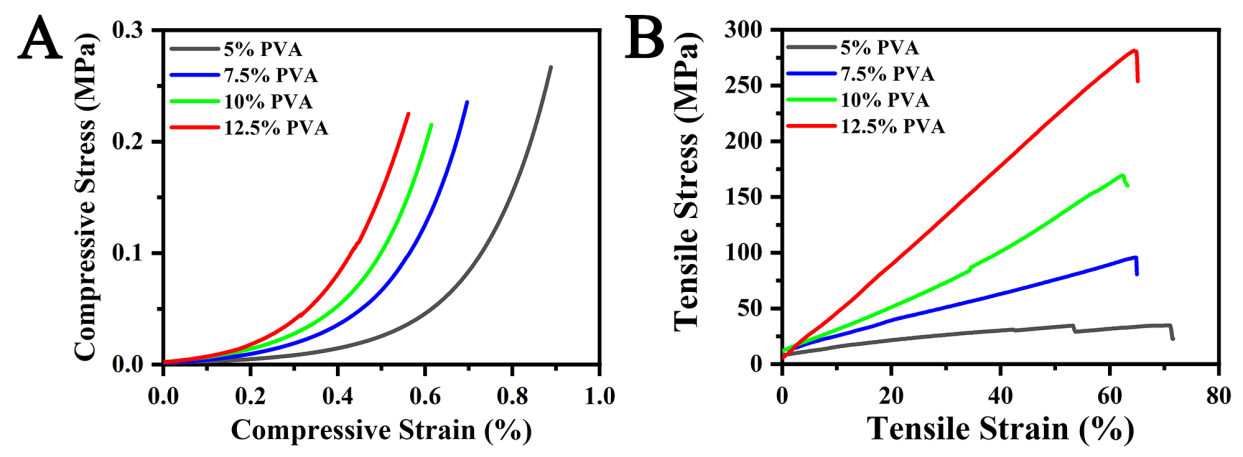


**Fig. S2** (A) The tested diagram between hydrogel compressive strain and compressive stress under the different reaction concentrations of PVA (wt 5%, 7.5%, 10% and 12.5%) of the prepared hydrogels. (B) The tested diagram between hydrogel tensile strain and tensile stress under the different reaction concentrations of PVA (wt 5%, 7.5%, 10% and 12.5%) of the prepared hydrogels.


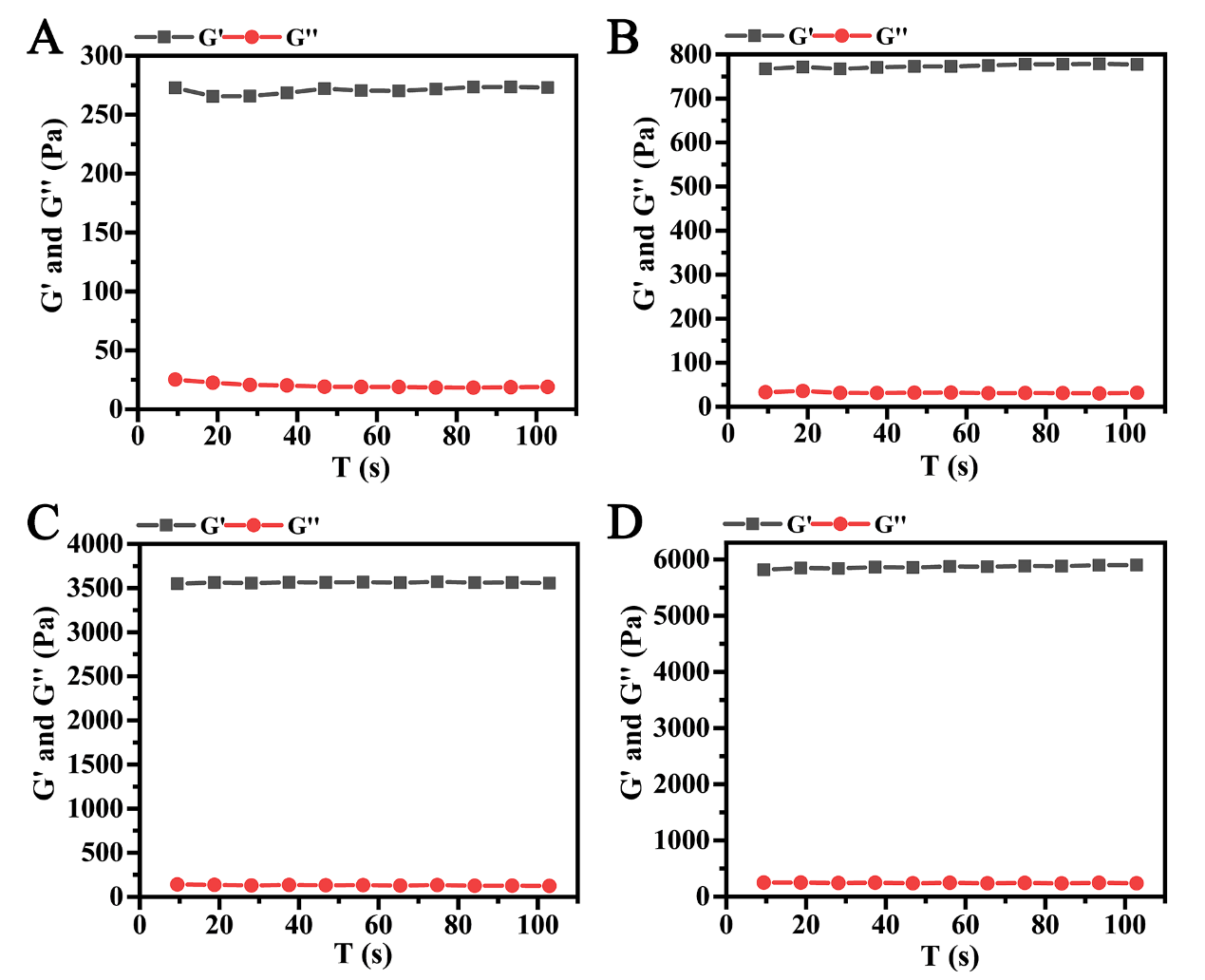


**Fig. S3** G' (Storage modulus) and G'' (Loss modulus) dependence vs time in continuous step strain measurements at the different reaction concentrations of PVA (A wt 5%, B wt 7.5%, C wt 10%, and D wt 12.5%) of the prepared hydrogels.


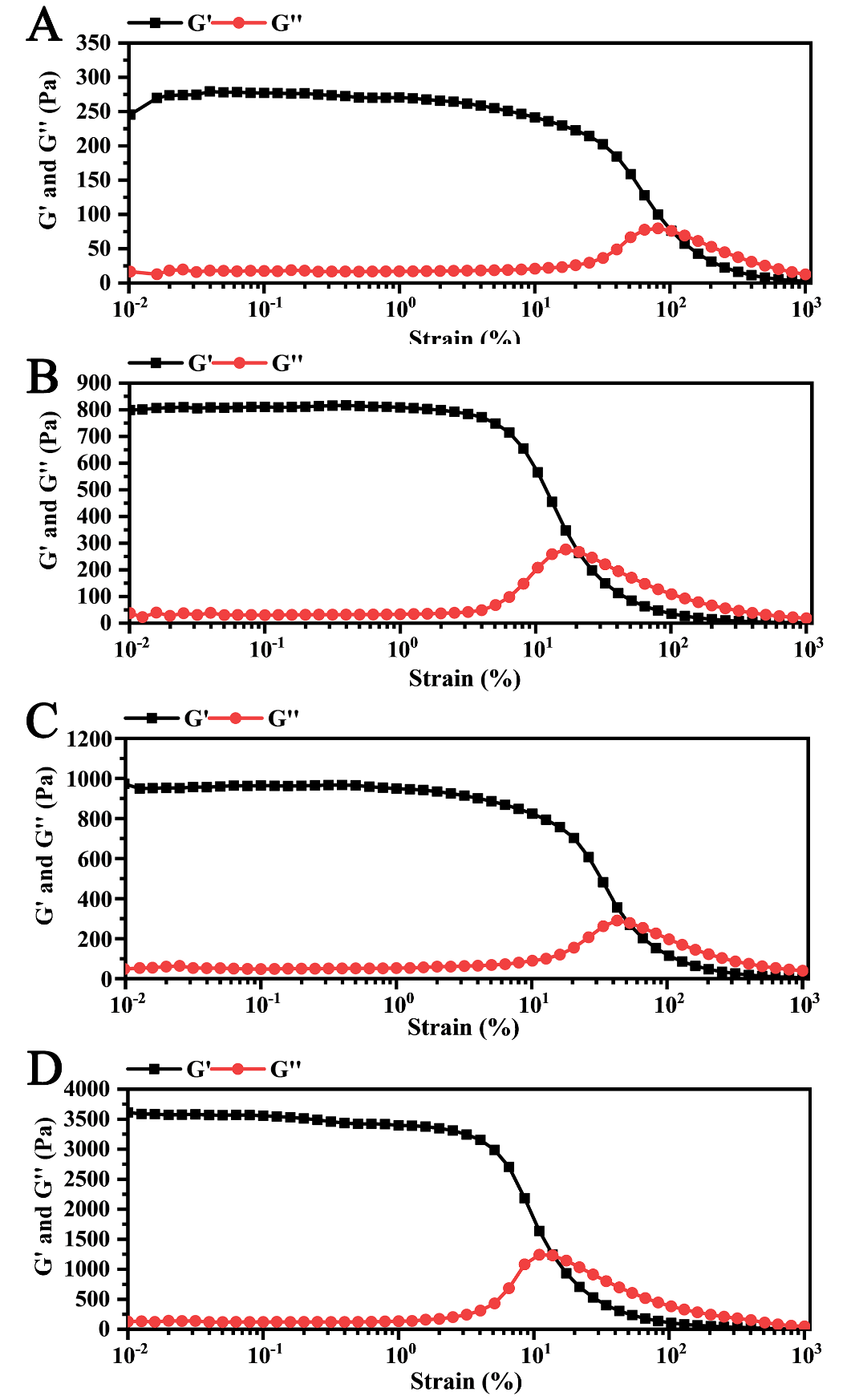


**Fig. S4** The strain amplitude sweeps of hydrogels with the different reaction concentrations of PVA: A wt 5%, B wt 7.5%, C wt 10%, and D wt 12.5%.

**
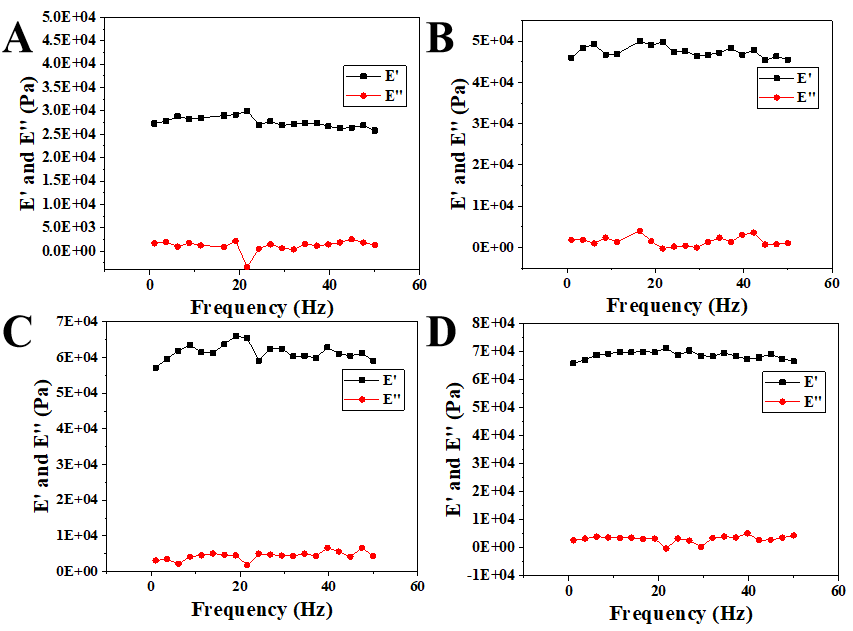
**

**Fig. S5** The test diagram of dynamic storage modulus (E') and loss modulus (E'') of prepared PVP@Ag NPs/CPH with different PVA concentrations: (A) 5 wt%, (B) 7.5 wt%, (C) 10 wt%, and (D) 15 wt%, from 0 - 50 Hz.

The E' of the hydrogels raises with the increasing of the PVA reaction concentration.

**
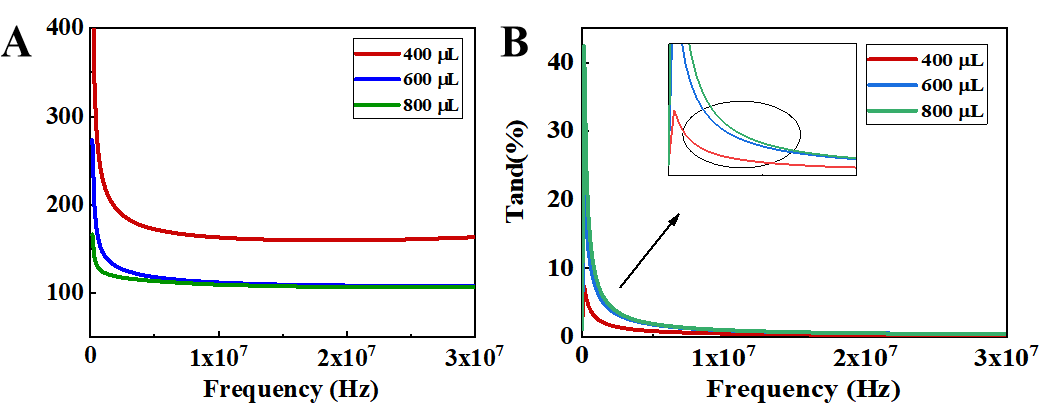
**

**Fig. S6** The dielectric constant of PVP@Ag NPs/CPH with different AN content: (A) is the real part; and (B) is the imaginary part of the dielectric constant.


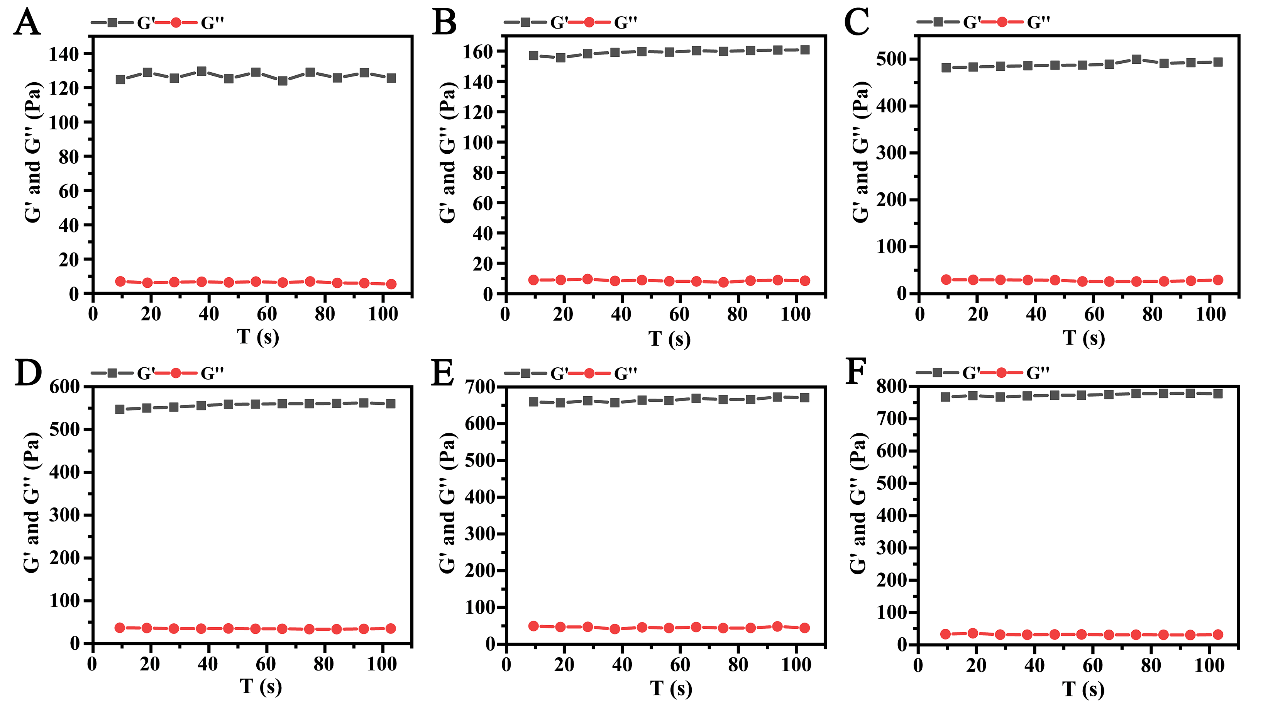


**Fig. S7** G' (Storage modulus) and G'' (Loss modulus) dependence of time in continuous step strain measurements for hydrogels with the different gelatin contents: (A) 0.5 wt%, (B) 1 wt%, (C) 1.5 wt%, (D) 2 wt%, (E) 2.5 wt%, and (F) 3 wt%.


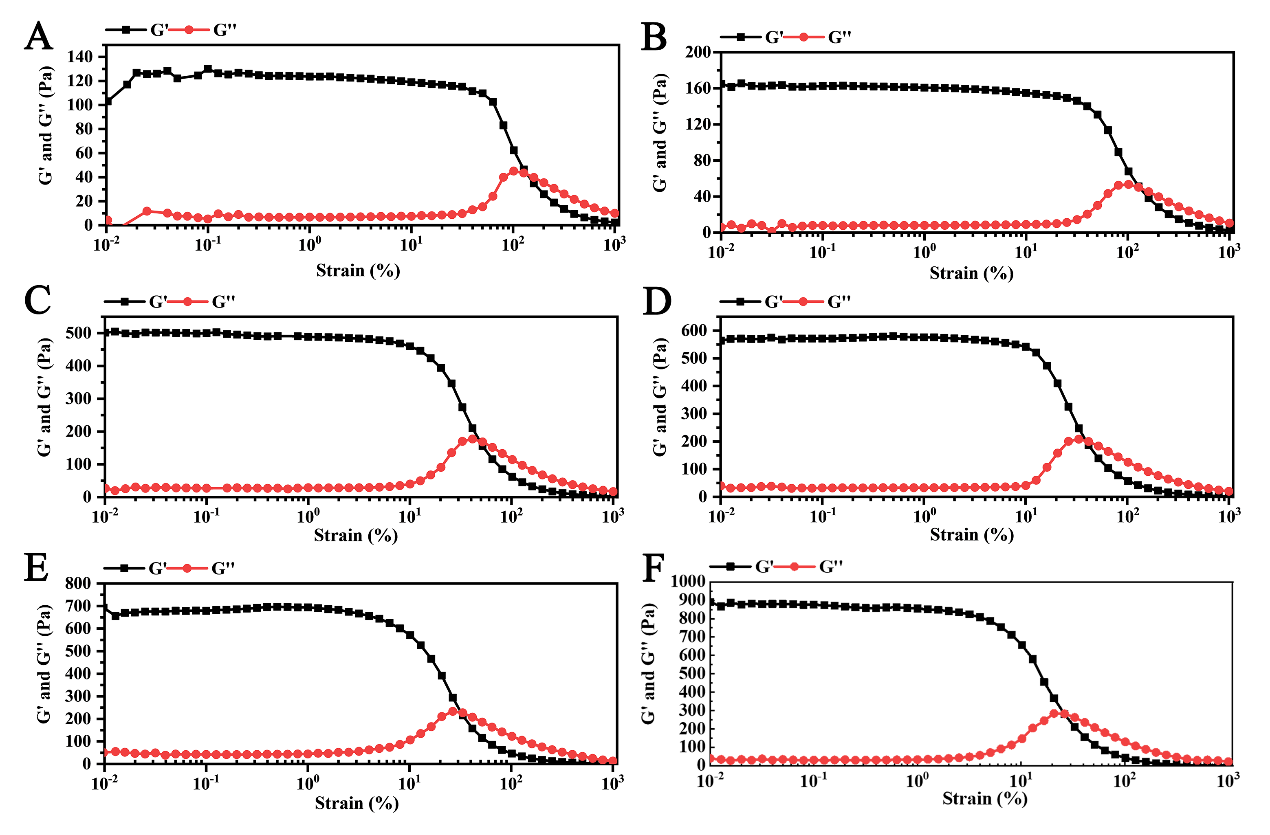


**Fig. S8** The strain amplitude sweep of hydrogels with the different gelatin contents: (A) 0.5 wt%, (B) 1 wt%, (C) 1.5 wt%, (D) 2 wt%, (E) 2.5 wt%, and (F) 3 wt%.

**
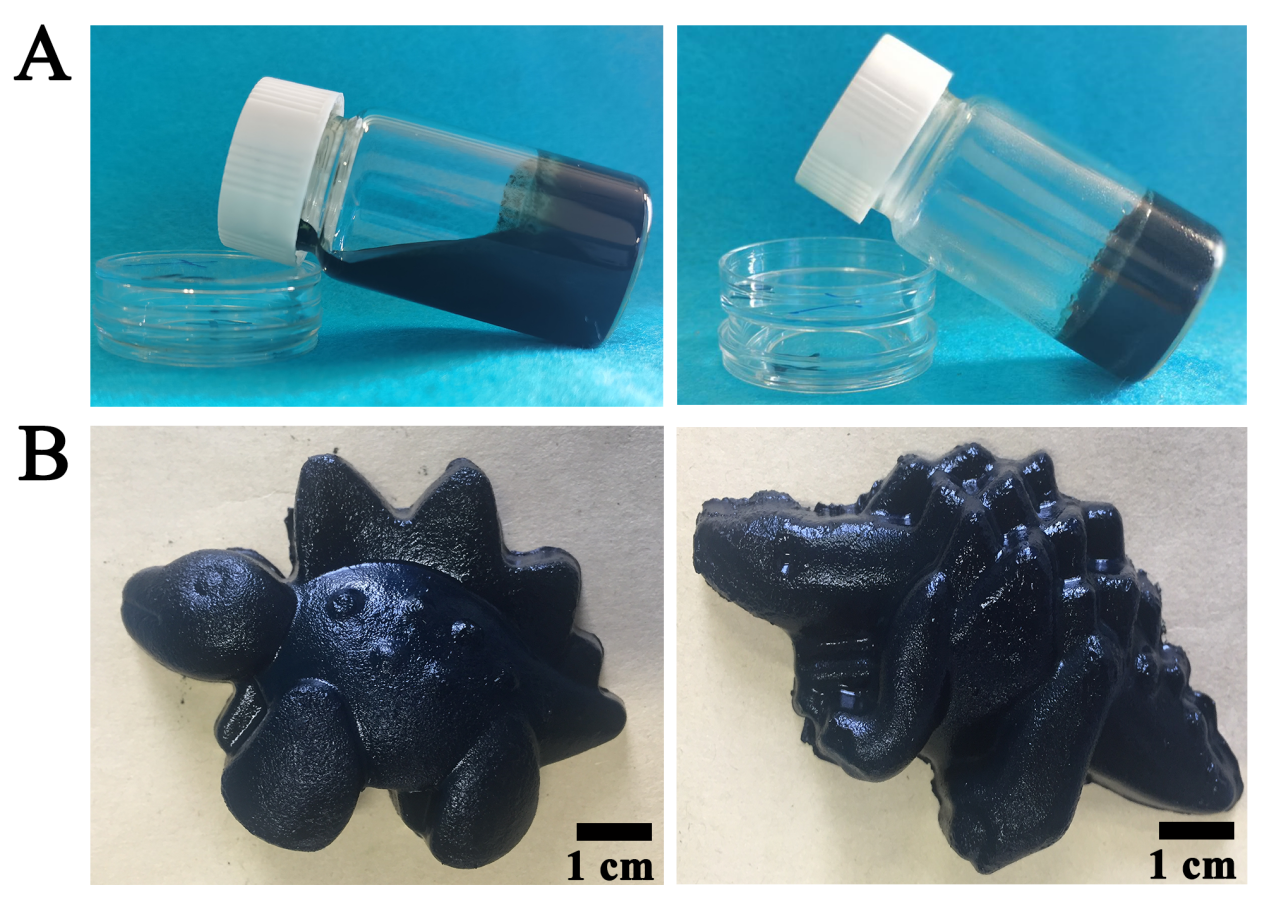
**

**Fig. S9** (A) Images of the flowing Ag NPs/CPH sol (left) without freeze-thawing, and the immobile Ag NPs/CPH gel (right) after freeze-thawing at room temperature. (B) Images of the Ag NPs/CPH material with different shapes, using the same molds for freeze-thawing.


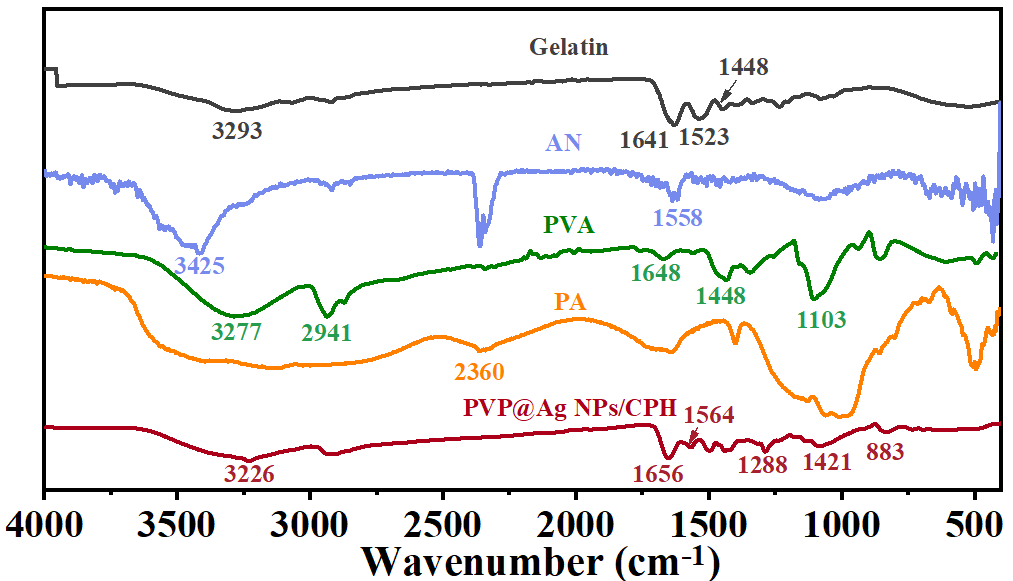


**Fig. S10** FTIR spectra of gelatin (black line), AN (purple line), PVA (green line), PA (orange line), and PVP@Ag NPs/CPH (red line), respectively.

**
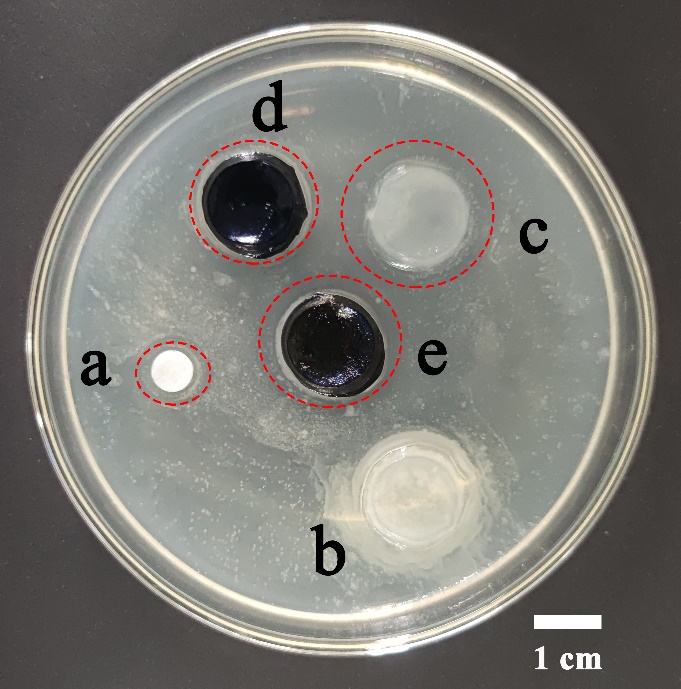
**

**Fig. S11** Photo of the antibacterial plate experiment (S. aureus) for the nanocomposite hydrogel: a. Ag NPs group, b. PVA and gelation group, c. PVA, gelatin and Ag NPs group, d. CPH group, and e. Ag NPs/ CPH group.

Both Ag NPs/ CPH group and gelatin and Ag NPs group have obvious inhibition zones, as well as the Ag NPs group. It shows that the antibacterial effect of Ag NPs plays a leading role;

The CPH group has a smaller inhibition zone, which may be derived from the surface antibacterial effect of polyaniline;

PVA and gelation group does not have any antibacterial zone, and even the bacteria grow up near the patch;

It indicates that the main antibacterial effect of Ag NPs/CPH comes from Ag NPs, while polyaniline plays an auxiliary antibacterial effect. And other components such like PVA and gelation have no antibacterial activity.

**
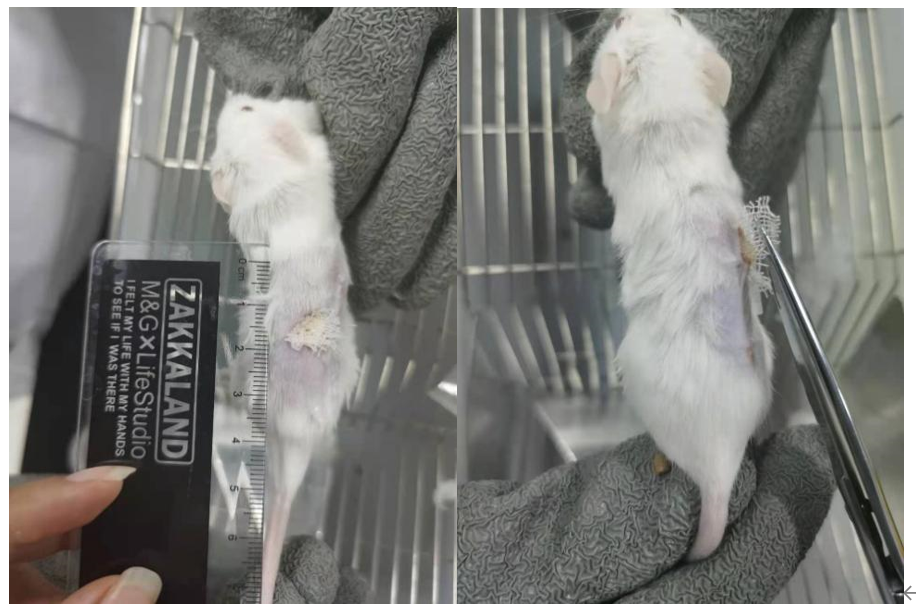
**

**Fig. S12** Photos of gauze adhesion near the wound of the mice in the control group.
